# Cook County Hospital

**Published:** 1869-10

**Authors:** Curtis T. Fenn


### Hospital Reports.

ARTICLE I.—*Cook County Hospital.* Reported by CURTIS T. FENN, M.D.

THE number of fractures in the Surgical Department of Cook County Hospital is sufficient to show that these injuries are deserving of special attention. Add the cases likely to be consigned to other hospitals, or conveyed to the dispensaries, as well as those which fall to private hands, and the whole number whose broken bones are splinted in Chicago daily would give several surgeons exclusive employment. This great field is comparatively neglected. While the most diverse views are taught, a deplorable lack of observation exists among students at large. A minute knowledge of anatomy and pathology is required to observe correctly the phases of a fracture from beginning to end. Few are found to acquire it. It is impossible, therefore, to escape error in the diagnosis and treatment, to say nothing of having the tact and patience so necessary in the management of fractures.

The importance of this branch of surgery demands it. They are all grave injuries, not only in respect to the deformity, the loss of valuable time, and the deterioration of health to the patient on the one hand, but also in respect to the liability of the surgeon on the other. The speediest union, and the least deformity attainable in a given case of fracture, will be had by the practitioner who has brought love of the truth to the study of healthy nutrition and repair.

The hospital furnishes a noble field for clinical study in the direction indicated.

The following is a statement of the fractures admitted during the year ending July 31, 1869, with reference to the time when they occurred, and the part injured:

August — 1 leg.
September — 1 leg, 2 forearm.
October — 1 leg, 1 forearm, 1 arm, 2 jaw, 1 rib.
November — 3 leg.
December — 2 leg, 2 thigh, 1 forearm.
January — 4 leg, 1 patella, 1 arm, 1 rib.
February — 2 leg, 1 patella, 1 clavicle.
March — 1 leg, 1 arm.
April — 1 leg, 1 forearm, 2 rib.
May — 2 leg.
June — 5 leg, 2 arm, 1 scapular, 1 jaw, 1 skull.
July — 1 foot, 4 leg, 3 thigh, 2 clavicle, 1 rib.

The list excludes cases of railroad injury in which death or amputation followed. It will be observed that of 58 cases, 16, or over 27 per cent., took place in the months of December, January and February, while 21, or over 36 per cent., occurred in the months of June and July.

The cases existing in the Hospital, July 31, are enumerated below, with so much of their history as was accessible.

1. Fracture of left humerus at upper end of middle third, March 24, 1869. Admitted June 4. Swedish emigrant, student, aged 26, of good habits. His injury occurred on shipboard, one week before arrival in New York. While walking on the deck of the vessel he slipped and fell. The fracture was dressed rudely. It was redressed and straightened, March 31, in New York. While on his way to Chicago, May 1, he was jostled against the wall, in a crowded car, and his arm refractured. On admission considerable deformity existed. A straight splint was applied, and the case recorded as one of delayed union. The splint was removed June 24, the indications of complete union being satisfactory. Two days later, while the patient was walking out by permission, he fell, and in putting out his hands to save himself, again reproduced the fracture. There was no crepitus, but marked deformity and mobility at the spot affected. A straight splint was reapplied without correcting the deviating bone wholly. He was discharged August 2. Union firm. Period, 129 days.

2. Fracture of right leg, tibia at upper end of middle third, fibula at lower end of middle third, May 14, 1869. Admitted same day. Born in Ireland; laborer; aged 40; of irregular habits. A curb-stone fell over on his side. Injury accompanied by marked deformity. A box splint was used the first three days; but, getting a chance in the night, he kicked it off, allowing the deformity to return. The fracture was readjusted in the morning, and a plaster of paris splint applied. Removed the 42nd day. Good union. Considerable tenderness. Cold douche daily. Limb kept bandaged, and at rest in an elevated position. Shortening one-fourth of an inch. Discharged August 11. Period, 89 days.

3. Fracture of right scapula at neck, June 17. Born in Sweden; farmer; aged 19; habits good. He fell from the back of a mule, in the field, striking with his shoulder. Treatment, support to the arm. Union prompt. A depression under the acromion remained, causing a slight deformity. Discharged the 52nd day.

4. Fracture of right leg, tibia at lower end of middle third, fibula at upper end of middle third, June 18. Admitted same day. Born in Ireland; teamster; aged 23; habits good. In wrestling he was thrown to the ground, the weight of his opponent falling across his leg. Deformity marked. A plaster of paris splint was applied at once, and retained five weeks, when it became uncomfortable and gave place to another for six days. Union then perfect. Shortening one-half an inch. Discharged the 53rd day.

5. Fracture of left superior maxilla-alveoli from second incisor to third molar; completely detached, June 24. Admitted same day. Bohemian; laborer; aged 55; habits good. He fell in a fit on the sidewalk, striking with his face. Displacement downward and inward. Teeth firm. A lacerated and contused wound over left malar bone and down across seat of fracture. No retentive apparatus was used, other than the four-tailed bandage. The external wound cicatriced. Small bits of necrosed bone escaped through the gums about the 21st day. Soon after, an abscess formed and broke over the recent cicatrix. The opening continued to discharge pus. The 37th day there was no union. The fragment was held in place only by mucous membrane. A

probe passed horizontally in at the external opening crossed the antrum, touching carious bone. Removal of the loose portion of the dental arch having been decided upon, the patient went out August 2, to avoid the operation.

6. Fracture of left tibia at lower end of middle third, May 28. Admitted June 9. Born in Michigan; servant girl; aged 28; habits good. She fell down a flight of twelve steps. No deformity save swelling and ecchymosis. Sat up and bathed the part with liniment the first twelve days. On admission a box splint was used for six days, then plaster of paris till the 48th day, when union was perfect. No shortening. Discharged August 4. Period, 68 days.

7. Fracture of left femur, complicated with compound comminuted fracture of left patella, June 29. Admitted same day. German; laborer; aged 21; habits good. He fell from the new city hall, two stories, without obstruction, while carrying a hod of mortar. Contusion of face frightful. Deformity of thigh marked. Patella punctured upon the center of anterior surface, and broken into four pieces. The opening admitted a finger to be passed into the joint. Synovia escaped. A wet compress was kept on the knee, and a weight of eighteen pounds fastened to the leg. There was apparently perfect bony union of the patella at the end of four weeks, without anchylosis. The bone was one and a fourth inches broader, and one inch longer than normal. The femur united readily, with considerable displacement. Shortening one and a fourth inches.

8. Fracture of right leg at middle of middle third, July 1. Admitted same day. German; laborer; aged 22; habits good. Injury caused by his jumping off a wagon which was in rapid motion. Deformity marked, the crest of the lower fragment of the tibia projecting nearly through the integument. It could not be reduced by extension. A plaster of paris splint was used, carefully avoiding compression upon the projecting fragment for fear of ulceration. The first splint was removed the third week. The second was removed August 10. Union perfect. Considerable deformity. Shortening one inch.

9. Fracture of right femur at neck, both within and without capsule, July 2. Admitted same day. Born in England; carpenter; aged 25; habits good. He fell, with scaffolding, twentyfive feet, striking with his hip across a tool box. Symptoms: Helplessness, slight eversion, crepitation, rotation of trochanter, with a shortened radius; the head felt at rest through the spare gluteal coverings when the trochanter was moved. Three-eighths of an inch shortening. Treatment: A pad was placed beneath the trochanter, and an eighteen pound weight attached to the leg. August 8 the dressing was removed. Union perfect. Shortening slight.

10. Fracture of right clavicle at inner end of outer third, July 3. Admitted July 10. Born in Ireland; an express driver; aged 18; habits defective. Thrown from a wagon, he struck the cobble stones with his shoulder. When admitted he was wearing Hamilton's dressing. Marked deformity existed without mobility. Redressed with adhesive straps. No compress. United readily, with shortening and projection. Discharged August 7. Period, 34 days.

11. Fracture of left eighth and ninth ribs at middle, July 3. Admitted July 16. Born in Ireland; laborer; aged 40; habits good. Fell across a ditch-box. Treatment: support by bandage. Discharged Aug. 2. Period, 30 days.

12. Fracture of right femur two and a half inches above articular surface, complicated with dislocation of condyles into the popliteal space, July 3. Admitted same day. Born in Ireland; peddler; aged 54; habits good. He fell through a trap door in the third story, striking upon the lower floor. Deformity well marked. Chloroform was administered and the dislocation reduced by manipulation and extension, an operation of some difficulty. Systematic extension and counter extension were applied to the leg. Union perfect the 43rd day. Shortening, one-half inch.

13. Fracture of left leg at lower end of middle third, July 10. Admitted July 11. Born in Norway; bricklayer; aged 42; habits good. He broke through a hatchway and fell into the hold of a propeller while carrying his trunk on board. Marked deformity. A plaster of paris splint was used at once. Reapplied the 17th day. Ununited on the 52nd day. Shortening one-half inch.

14. Fracture of left leg at lower end of middle third, July 12. Admitted same day. Born in Ireland; laborer; aged 50; habits good. He stepped through an old barn floor. A plaster of paris splint was applied for the first dressing. After the gypsum had set, it was found that the bandage was too tight. Removed the second day, on account of pain. Reapplied. Union the 37th day.

15. Fracture of right clavicle at middle of outer third, July 13. Admitted July 15. Born in Ireland; lumberman; aged 23; habits good. He fell from a scaffold, fourteen feet, striking a plank with his shoulders. Dressing by adhesive straps. No apparent deformity. Shortening three-fourths of an inch.

16. Fracture of right leg at lower end of middle third, July 21. Admitted July 22. Born in Ireland; laborer; aged 22; habits good. Received a horse kick. Deformity marked. Left tibia subject of old disease, resulting in hypertrophy; lengthening manifest. A plaster of paris splint was applied to the fractured limb at once. Considerable deformity will remain. The leg bows out, while the sound limb is abnormally straight. Disparity of length, one and three-fourths inches.

17. Fracture of right first metatarsal bone, just back of head, July 21. Admitted July 29. Bohemian emigrant; aged 23; habits good. His foot was caught between the cars. Swelling and ecchymosis. Treatment: cold wet compress. Union the 28th day.

18. Fracture of left leg at upper end of lower third, complicated with deep laceration of the inner side of calf, above and along side of fracture, July 28. Admitted same day. Born in Germany; painter; aged 23; habits good. He fell under a street car in motion. Two wheels passed over him. The first produced the fracture, the second the flesh wounds. Considerable contusion of the outer aspect of the calf. Deformity marked. The limb was extended and a support of plaster of Paris fitted to exclude the laceration. This was removed the third day on account of inflammation, and a poultice of flaxseed applied, the limb resting on a pillow. On the twelfth day, sloughs had separated, leaving deep cavities in place of the lacerations. Discharges copious from exuberant granulations. Cerate dressing used. The swelling of the foot disappeared. He emaciated rapidly, settling down in bed. On the 19th day he had a chill; pulse rose to 114; considerable uneasiness; imperfect sleep; tongue coated, thin, white; tenderness over abdomen; tympanites. His fever continued; emaciation progressed; the wounds discharged unhealthy pus; the loss of tissue extended, the integument becoming undermined and melting away at the margin of the wounds. Communication with the bone to a wide extent became evident. He sank lower in bed. Profuse perspiration bathed his body through the day. At night he slept, under the use of morphia. He complained very little. At length all strength failed. The ulcers grew black and gangrenous, and he died on the 32nd day.

19. Fracture of left temporal bone at upper part of temporal ridge; compound; June 21. Admitted same day. Born in Ireland; soldier; aged 31; habits intemperate. Received a horse-kick, the toe calk penetrating the skull. Opening through the integument $1\frac{1}{4}$ inches long; through bone $\frac{1}{2}$ inch long. Small spiculæ picked out. Duramater unbroken. Ice was applied at once. On the 25th day meningeal inflammations appeared. Treatment: veratrum viride and belladonna. The wound closed rapidly. He went out the 34th day, at his own request. Getting on a spree, he returned in two days with a smart attack of erysipelas. Met by the tincture of chloride of iron.

Of this group of cases, No. 1 is remarkable as illustrative of frequent causes of delayed union, namely: anæmia, insalubrity of emigrant traveling, mental dejection added to disturbance at the seat of fracture by want of care in handling. No. 2 may show the effect of too much handling, in the irritability remaining. No. 3 is a rare case. No. 7 is an example of unmistakable bony union in a comminuted compound fracture of the patella. So considerable a shortening of the femur would admit of close approximation in spite of the tonic contraction of the extensor muscle. No. 9 presented a case of difficult diagnosis as well as rarity. No. 12 is remarkable on account of the complication and the excellent result obtained, as in No. 16, on account of the unhappy dissimilarity of his legs. No. 18 is one of those cases which are rare upon the whole, yet deplorably frequent in respect to their forcible admonitions and inevitable result.

Case 1. — *Abdominal Hæmorrhage.* — L. S., a German woman, small, aged 43, had been an inmate since June 19, 1868, for chronic hypertrophy of the womb, accompanied by retroversion, cervicitis, and ulceration, giving a history of instrumental delivery, frequent confinements, and hard work at service. For sixteen years, she was complaining not bearing children, having all the symptoms commonly present in such cases, culminating in hysteria. For a time after her admission, treatment was unavailing. Finally, about four weeks previous to her death, Dr. Jones amputated the anterior lip of the cervix. The cut surface was tough and fibrous, having a lateral diameter of one inch; vertical diameter, three-fourths of an inch. Her condition, after the operation, was generally improved; her menses came on at the regular period, instead of within it, as before, and continued for the usual time; the hæmorrhage from this source was excessive; however, controlled by gallic acid. Two days previous to the occurrence we are to describe, it had ceased altogether, and her condition seemed to promise a cure. The wound of the operation was at this time nearly healed. August 16, 1869, she was waked, at 2 o'clock in the morning, by profound pain in the abdomen, referred to the hypogastrium. She remained on her back through the day, in a state of complete collapse. Pain was controlled by opiate enemata. In twenty-four hours she died. *Autopsy thirty-six hours after death.*—Rigor mortis; considererable layer of fat beneath the integuments; atrophy of mammary glands; transverse colon black and distended; dark fluid escaping from the cavity of the abdomen, containing grumous particles. A pint of this fluid was taken out by sponges. The colon contained a pint and a half of the same liquid; the meso-colon was widely and generally infiltrated with extravasated blood, presenting a surface of coagulum, when the sub-peritoneal connective tissue was cut. The liver and spleen were both devoid of blood; the uterus was normal in size, deviating slightly to the left side; the cervix was indurated and hypertrophied; the ovaries were atrophied, containing many cysts of small size; deviations and adhesions of the Fallopian tubes existed on both sides. No signs of inflammation of the pelvis, the extravasated matter in the abdomen being confined to the hollows on either side of the spinal column. The real lesion was not discovered. The blood was found extravasated. There was room for speculation as to its origin.

Case 2. — *Apoplectic Aphasia.* — P. N., aged 55, born in Ireland, a laborer, was admitted July 1, 1869. No previous history of his case exists save a single statement to the effect that some time before his admission, he fell, suddenly, and lay for awhile insensible. He was partially paralyzed on admission, and dumb, though he understood. Paralysis of the sphincters was total; the right side was nearly helpless, the left enjoyed greater freedom. His face and larynx were not affected; his inability to speak was evidently of another sort. He had double cataract. His intelligence gradually failed, and he lay, finally, for several weeks, in a state of complete lethargy. Toward the last, he had slight tonic convulsions; on the sixth day they were repeated with greater violence; on the seventh day, August 25, he died.

*Autopsy twelve hours after death.* — Strong adhesions of the thickened duramater; about half a pint of serous fluid was found within the cranium. When the brain was inverted and lay upon its upper surface, the left anterior lobe seemed retracted; a distinct sense of fluctuation was obtained. On opening the lobe, it was found generally softened, containing, in the centre, a circumscribed pultaceous mass, the size of a walnut. The basilar artery was nearly occluded; its walls pearly and cartilaginous in texture. The same condition was found more or less extensive in the cerebral arteries of either side. The spinal cord was normal. No further examination took place. There can be little doubt as to the origin of the aphasia.

Case 3. — *Diffused Aneurism of the Aorta.*—J. L., aged 33, born in Ireland, sixteen years in this country, a farmer of previously sound constitution, was admitted August 16, 1869, and the case recorded as malarial cachexia. In the fall of 1868, he left a farm in central Illinois, to work on the Union Pacific railroad. In February, 1869, he had several chills, for which he took quinine. In March, he quit work altogether, owing to pain between his hips. It was not constant. He had an uncomfortable bed and bad food, and consequently referred his pain to the exposure he endured. He had fever all the time, but no sweating or pain, other than that mentioned, which was worse in the morning. He continued to suffer from fever, loss of appetite, thirst, costiveness, and weak digestion; being able to eat only a little rice.

He rapidly emaciated. After arriving in Chicago, March 18, he gained, but as soon as the weather became warm he again declined. He complained of sweats, a dirty tongue, constipation, and great pain in making water — involving the left inguinal region — it seemed as if it would kill him. His urine contained considerable offensive sediment; the pain in his back grew in severity, affecting him most in the morning, and especially whenever he took a drink of water or tea; operations of oil, which he took to move his bowels, sometimes gave great pain in the left inguinal region; his testicles retracted — he had gonnorhœa the year before. On admission he was greatly emaciated; tenderness and tumefaction beginning over the spleen, extended into the inguinal region. Toward the last he had retention of urine. He sank under gradual aggravation of all the symptoms until August 29, when he died.

*Autopsy twenty-four hours after death.* — On opening the abdomen, dark, red discoloration marked the surface of the small intestines; the omentum was black, and adherent to parts in contact with it; black infiltration was general within the sub-peritoneal connective tissue in the walls of the entire hypogastric, left inguinal, and left lumbar regions. A huge oval mass filled the space described, limited only by the walls of the abdomen and the spinal column, extending upward to the diaphragm, and below to the cavity of the pelvis covered by the peritoneum of the lateral and posterior parietes. The hand readily broke into the tumor; the central portion consisted of a coagulum. On turning this out, sheets of fibrine, beautifully laminated, peeled up, having a deeply-rippled surface from four to six inches in diameter; layer after layer of this substance came to view, growing tougher and paler toward the bottom. The base of the tumor was found in front of the second and third lumbar vertebræ. The bodies of these bones were bare, and eaten away on either side to the depth of half an inch. A smaller division of the sack existed to the right of the column, furnishing the same proportion of fibrine and coagulum. The liver appeared about normal; the spleen was small and crowded up; the intestines were empty and crowded up to the right side; the bladder was distended; the left kidney was elongated, and its pelvis filled with urine. Owing to a somewhat unguarded dissection of the tumor, its exact origin was not made out. The aorta seemed normal immediately above and below the eroded vertebræ. The mouth of the aneurism was not found. The central coagulum, fibrinous, stratified walls, and wide extravasation, proved incontestibly the nature of the case.

CASE 4.—*Rupture of Aortic Semi-lunar Valves.*—J. C., colored, aged 56, a roistabout, was admitted August 28. Two weeks previously, he had felt a sharp pain, with a sensation of something giving way, about his heart. He was at the time, with others, lifting a heavy piece of machinery, the whole strain of which came upon him. Immediately after this exertion, he felt difficulty in breathing. On admission, dyspnœa was marked ; a blowing sound was heard over the base of the heart. Two days later he died suddenly, after rising up in bed to receive a drink of water he sank back and expired.

*Autopsy.*—The heart was small and rather flabby ; its ventricles were full of fibrine adhering closely to the walls ; two of the aortic valves were ruptured ; two large, irregular rents, whose united calibre was almost equal to that of the vessel, existed in the membrane of each ; the borders were intact. The apparatus was thus rendered totally insufficient. Considerable attenuation of the remaining valve, amounting to atrophy is observable. The lungs were œdematous. No other lesion was demonstrated.

